# Multifaceted Work-to-Life Negative Spillover and Depressive Symptoms among Working Women: The Moderating Effect of Social Activities Satisfaction

**DOI:** 10.3390/ijerph191811572

**Published:** 2022-09-14

**Authors:** Jeong-Hyun Nam, Soo-Hyun Nam

**Affiliations:** 1 Department of Korean Medicine, School of Korean Medicine, Pusan National University, Yangsan 50612, Korea; 2 Department of Nursing, Hallym Polytechnic University, Chuncheon-si 24210, Korea

**Keywords:** work-to-life negative spillover, depressive symptoms, mental health, social activities satisfaction

## Abstract

This study aims to examine how work-to-life negative spillover is associated with depressive symptoms among working women and to explore moderating effect of social activities satisfaction on the relationship between work-to-life spillover and depression. This was a secondary data analysis from a sample of 2869 employed women from the 7th Korean Longitudinal Survey of Women and Families. The results showed that work-to-life negative spillover was positively associated with depressive symptoms. Additionally, there was a significant moderating effect of social activities satisfaction on the relationship between work-to-life negative spillover and depressive symptoms (β = 0.176, *p* < 0.05). It was found that the low social activity group showed fewer depressive symptoms induced by the negative work-to-life spillover than the high social activity group. Based on the results of our study, effective strategies and policies for work-family compatibility and interventions aimed at reducing the work induced stress and depressive symptoms are recommended.

## 1. Introduction

Employees’ desire to maintain balance between work and non-work is a global consensus. Individuals who feel that their work provides positive influence in their non-work-related areas may experience increased sense of well-being and motivation [1]. Especially in South Korea, which has the third-highest number of working hours among the OECD countries after Mexico and Costa Rica [2], workers are exhausted from work ethics that are often self-sacrificing and consist of long working hours and life without “going home” [3,4]. Overtime work with high intensity used to be a norm in developing Korea [5]; however, there has been a change in society, reflecting workers’ desire for work–life balance [3]. If a worker ascribes their unhappiness to exhaustion at work, emphasizing old work ethics such as self-sacrifice, enduring long working hours without stipulated rest time or vacation [5,6] might not be adequately persuasive to them. According to the results of the ‘2019 Social Survey’ by the National Statistical Office, among Koreans aged 19 and older, those who prioritize work–life balance accounted for 44.2% of the responses [7].

Work-to-life negative spillover occurs when an individual encounters strong negative experience and emotions, which are carried over from work to home, and it has been found to be associated with general health outcomes and individual well-being [8,9,10,11]. Previous studies suggest the work-to-life negative spillover is significantly associated with multiple health outcomes. One meta-analysis reported that work-to-family spillover has negative effects not only on physical health but psychological problems such as depression, stress, and anxiety [10]. Longitudinal associations were found between work-to-life negative spillover and increase in chronic diseases [12]. Other researchers, on the contrary, stated that women with multiple roles at work and home may have opportunities to utilize more diverse social resources [13]. Therefore, no consistent conclusion can be drawn from the existing literature in regard to the relationship between work-to-life spillover and its effects on individuals.

Social activities satisfaction, defined as self-satisfaction from social engagement that seeks emotional pleasure and relaxation, is an important capital for mental health and subjective well-being [14]. The Korean Longitudinal Survey of Women and Families defined social activities as “An activity that seeks emotional freedom, relaxation, and pleasure, which is carried out during one’s free time, excluding working hours and time for physiological needs (sleeping time, meal time, etc.) out of a 24 h day”. A multi-center study conducted across the world, including Asia, Africa, Europe, and America, reported that decrease in social activities, such as gathering with neighbors, friends and family, and activities for pleasure and rest, were significantly associated with psychosocial strain during home confinement during the COVID-19 period [15]. In a prospective cohort study, social activities were a protective factor against depressive symptoms. [16]. A longitudinal study also showed that leisure time and physical exercises diminished poor health outcomes brought by negative spillover [11]. Similarly, absence of social activities increases one’s vulnerability to psychological distress and lowers individual’s life satisfaction [17]. Those with high satisfaction in social activities could alleviate difficulty in both work and life and improve their potential to cope with it [18].

Thus, it is necessary to look at multiple aspects of work-to-life negative spillover, to analyze the impact of women’s mental health as well as on how to mitigate variables affect this relationship. Few studies have investigated effects of multidimensional effects of work-to-life spillover on depressive symptoms using the national representative data of Korean working women. Furthermore, there is—to the best of our knowledge—no study investigating the moderating effect of social activity satisfaction in the relationship between work-to-life negative spillover and psychological problems. Finding the interrelationships of these factors may contribute to improving mental health of working women, by providing organization managers and policy makers with useful insights on planning employees’ mental health care. Thus, this study aims to (1) explore how work-to-life negative spillover is associated with depressive symptoms among working women in South Korea, (2) examine moderating effect of social activities satisfaction on the relationship between work-to-life spillover and depression.

## 2. Materials and Methods

### 2.1. Data Collection

**Figure 1:**
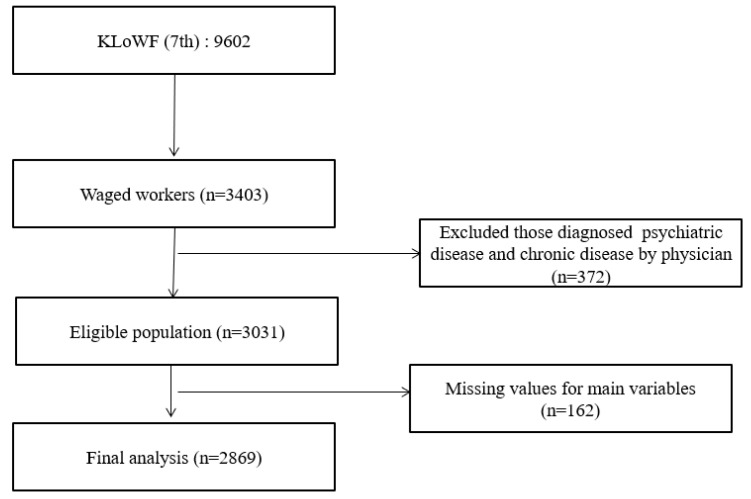
Flow diagram of inclusion and exclusion of participants. **Note**. KLoWF, Korean Longitudinal Survey of Women and Families.

We obtained data from the Korean Longitudinal Survey of Women and Families (KLoWF) collected between 2017 and 2018. The KLoWF is a panel survey conducted by the Korean Women’s Development Institute every two years since 2007. It used a stratified multistage random sampling design for representing sample of Korean women from a total 9620 individuals between the ages of 19 and 64. Computer-assisted personal interview system was used to conduct surveys and trained interviewers conducted face-to-face interviews. All of consent forms were obtained prior to participation and since the KLoWF is a national public data, free download is available for the purpose of research. The KLoWF survey consists of the questions about the household, including family relationship, household income, housing, and consumption; individual information such as age, education level, marital status, and health issues; and work-related section such as individual’s economic activity, level of income satisfaction, and policy of workplace’s maternity protection. In this study, we used the 7th raw dataset surveyed in 2018 in the KLoWF panel, a total of 9620 participants since it was the most recent (Figure 1). Work-to-life spillover, depressive symptoms and social activities satisfaction were collected through self-reported questionnaires answered by the participants.

### 2.2. Measurement Variables

#### 2.2.1. Work-to-Life Spillover

Work-to-life spillover was assessed according to items that ask how much current work affects individual’s life in aspects of the six following spillover types: rewards and vitality in life, recognition from family members, satisfaction with family life, influence of work on children, influence of work hours on fulfilling one’s domestic obligations, and influence of work schedule on family life. Each response was in the form of a four-points Likert scale as one of ‘strongly disagree’, ‘disagree’, ‘agree’ and ‘strongly agree’. To measure work-to-life negative spillover, positive questions are inverted as negative questions, and respondents with high scores on the Likert scale are indicated as having high work-to-life negative spillover; a score of 1 indicates ‘strongly disagree’, and 4 indicates ‘strongly agree’.

#### 2.2.2. Depressive Symptoms

We used the 10-item Center for Epidemiologic Studies Depression scale (CES-D-10: a short form of CES-D-20 developed by National Institute of Mental Health studies) to assess depressive symptoms. The CES-D-10 has been a widely used instrument for the identification of depression with high reliability and validity. This scale has been proved not only in classifying participants with depressive symptoms with 91% sensitivity and 92% specificity but also as an excellent alternative to the original CES-D 20 as it contains the exact underlying factors of the original one [19]. The frequency of each item regarding depressive mood or symptoms in the past week on a 4-point scale was attained. A score of zero indicates that depressive symptoms have been experienced less than once during the past week, a score of 1 indicates one to two days, 2 refers 3–4 days, and 3 refers more than 5 days. The scores range from 0–30 for CES-D-10, and we used cut-off value of 10 so that those who with a total score of CES-D-10 ≥ 10 were classified into a depressive group.

#### 2.2.3. Social Activities Satisfaction

Participants were asked to describe how much they had been satisfied with the following domains for the purpose of rest and pleasure; hobbies and leisure activity, learning and self-developmental activity, social activities with friends, relatives, co-workers, and neighbors, volunteer activity, religious activity. The respondents with high scores are indicated as individuals who are highly satisfied with social activities calculated as 1 to 7 scores (1; very unsatisfied, 2; unsatisfied, 3; a little dissatisfied, 4; normal, 5; a little satisfied, 6; satisfied, 7; very satisfied).

#### 2.2.4. Other Variables

The socio-demographic variables and other information including age, education, income satisfaction, marital status, self-rated health obtained were as follows. Age (≤30, 30–39, 40–49, 50–59, and ≥60), education level (elementary school, middle school, high school, postgraduate), marital status (single, married, separated, divorced, or widowed), and income satisfaction (satisfied, moderate, dissatisfied) were obtained. Subjective self-rated health was classified into two groups, ‘good’ or ‘bad’ according to the items confirmed in the survey.

### 2.3. Statistical Analyses

**Figure 2:**
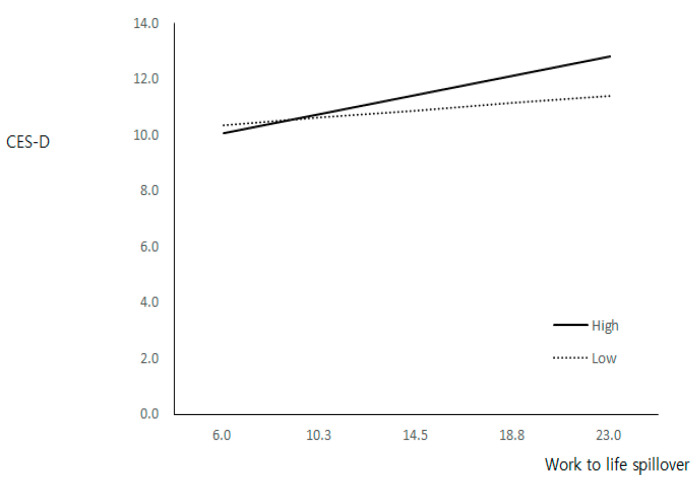
Interaction effects of social activities satisfaction on the relationship between work-to-life negative spillover and depression.

Socio-demographic characteristics and job as well as health-related variables of participants were analyzed using descriptive statistics. We then examined the differences in depressive symptoms according to main variables including age, education, income satisfaction, marital status, and self-rated health using chi-square tests. In the next step, we also checked the differences of the risk of depressive symptoms between non-depressed group and depressed group, based on type of work-to-life spillover. Multiple logistic regression analysis, which revealed and odds ratio (OR) with a 95% confidence interval (CI), was conducted to investigate the associations between work-to-life spillover and the depressive symptoms. Finally, the hierarchical multiple regression analyses were performed to test the effect of social activities satisfaction on depressive symptoms. General characteristics were added in the model 1. Work-to-life negative spillover and social activities satisfaction were entered in the model 2, 3, respectively. Finally, the interaction terms between work-to-life negative spillover and social activity satisfaction was added by adjusting confounding variable with independent variables. Based on the median of social activity satisfaction scores, the sample was divided into two groups; with high and low social activity satisfaction scores, and specifically confirmed the moderating effect of satisfaction in the relationship between work-to-life negative spillover and depression (Figure 2). In all tests, *p* values < 0.05 were considered to be statistically significant.

## 3. Results

### 3.1. Characteristics of Socio-Demographic Data

Table 1 shows general characteristics of the study participants. Participants’ mean age was 43.53 years (standard deviation/SD was 11.71). A total of 54.1% of all participants held postgraduate, while most were married (64.3%, 1845 participants). A total of 85.9% responded they were satisfied or moderate satisfied with their income level while 14.1% were dissatisfied. About 31.2% of participants responded that health status is bad. Looking at the results by dividing the constituent concept of negative work–life negative spillover, the proportion of participants who answered that “their work does not give rewards and vitality in life” was 6.6%, “their work does not give recognition from family members” was 13.6%, “their work does not give satisfaction with family life” was 14.1%, “their work does not give positive influence on children” was 29.7%, “their working hours do not give positive influence on fulfilling domestic obligations” 29.6%, “their work schedule does not give positive influence on family life” was 22.8%.

### 3.2. Association between Work-to-Life Spillover and Depressive Symptoms

Table 2 shows the prevalence of depression based on general characteristics and the type of work-to-life negative spillover. There was a statistically significant association between age and depressive symptoms (*p* = 0.04). Younger employees were more likely to be depressed than older employees. Marital status was also associated with participants’ depressive symptoms (*p* < 0.001). Participants who were the married more frequently expressed depressive symptoms than single people. There were no significant association between education, income satisfaction, and self-rated health with depressive symptoms. Except for work-to-life negative spillover related to recognition from family members (*p* = 0.162) and satisfaction with family life (*p* = 0.203), all types (vitality in life, influence of work on children, influence of work hours on fulfilling one’s domestic obligations, and influence of work schedule on family life) were associated with increased depressive symptoms (*p* < 0.001).

### 3.3. Association between Work-to-Life Spillover and Depressive Symptoms

Table 3 shows the results of multiple logistic regression analysis on the relationship between the existence of work-to-life negative spillover and depressive symptoms. Crude odds ratio was used as baseline and only considered the existence of work-to-life negative spillover and depressive symptoms while an adjusted odds ratio was obtained by adjusting age, marital status. For the group with existence of negative life spillover, the ORs (95% Cis) for depressive symptoms were 1.441 (0.83–2.51) for rewards and vitality in life, 1.374 (1.07–1.77) for influence of work on children, 1.590 (1.12–2.27) for influence of work hours on fulfilling domestic obligations, and 1.822 (1.26–2.63) for influence of work schedule on family life. After adjusting for age, marital status, the ORs (95% CIs) for depressive symptoms were 1.576 (0.88–2.82), 1.539 (1.19–2.00), 1.806 (1.28–2.54), and 1.969 (1.37–2.83), respectively.

### 3.4. Moderating Effects of Social Activities Satisfaction between Work-to-Life Negative Spillover and Depressive Symptoms

Hierarchical multiple regression analysis was conducted, as shown in Table 4 and Figure 2. In Model 4, the interaction terms between work-to-life negative spillover and social activity satisfaction were statistically significant (β = 0.176, *p* < 0.05). In other words, it was found that the moderating effect of social activities satisfaction was significant in the relationship between work-to-life negative spillover and depressive symptoms. As shown in Figure 2, depressive symptoms and work-to-life negative spillover increased in both the high and low social activity satisfaction groups, but the low social activity group showed less depressive due to the negative spillover than the high group. In sum, it was confirmed that the effect of work-to-life negative spillover on depressive symptoms was different according to the degree of social activity satisfaction.

## 4. Discussion

This study investigated the association between work-to-life negative spillover and depressive symptoms among Korean working women and further explored how social activities satisfaction attenuates this relationship. To the best of our knowledge, there is no country-wide study which investigates the moderating effect of social activity satisfaction on the relationship between work-to-life negative spillover and psychological problems.

Current data revealed that working women with existence of work-to-life negative spillover showed significantly high depressive symptoms. This result is in line with the findings of other studies reporting significant association between work-to-life negative spillover and depression [20,21,22]. A cross-sectional study conducted among U.S female employees showed that work-to-life negative spillover was associated with development psychiatric disorders such as anxiety, depression, and substance dependence [20]. Another study conducted among female workers in Switzerland reported that negative perception of spillover between work and life was related with depressive mood [21]. From these results, it can be seen that the degree of disturbance in life due to work and the lack of time allotted for housework or childcare are important factors that cause psychological burden on working women worldwide [22]. 

This result can also be explained through Korean women’s facing double burden of work and household duties [23]. Since Korean culture is rapidly transforming; from patriarchal to democratic, pre-industrial to post-industrial, and traditional to progressive, its current mental health issues regarding work–life balance may give useful insights on how women can maintain their mental health amid rapid socio-economic change. For example, the rate of Korean women’s participation in economic activities is increasing every year, reportedly up to 54.4% in 2019 [24]. However, a high proportion of working women in Korea still face discrimination in the workplace in regard to hiring, wages, promotion, and work assignments [25]. Although women’s economic activities have positive effects on the improvement of social role and status, on-going gender disparities in the workplace, despite new enforcement of the Sex Discrimination Act, still endanger women’s mental health [25]. Traditional gender roles such as domestic area of housework, parenting, and caregiving, are expected [26], which may induce a negative psychological burden and stress among working women.

From an organizational perspective, the imbalance between work and life leads to low job satisfaction, low productivity, and intention to change jobs [27]. Several countries, including the US and Canada, which have established policies such as flex-time work, remote work, or alternative work scheduling, have reported positive outcomes; employees’ increased work productivity [28,29,30,31,32], motivations, and decreased turnover intention. The sense of an organization’s fairness has also been reported as a protective factor to buffer the impact of work-to-life negative spillover on depression, especially among employees with low flexibility to manage their family life. It is necessary to create not only a workplace culture that enhances gender equity, but to improve family-friendly policy, securing work and life balance. Improving work–family compatibility policies such as shortened work during the childcare period, workplace childcare facilities, parental leave, parental leave for husbands, and childcare expenses support are necessary. Intervention aimed at reducing work-induced stress and depressive symptoms also needs to be implemented to improve Korean working women’s mental health.

Our study demonstrates that the impact of work-to-life negative spillover on working women’s depressive symptoms depends on the degree of satisfaction with social activities. Depressive symptoms and work-to-life negative spillover increased in both the high and low social activity satisfaction groups, but the low social activity group showed less depression due to the negative spillover than the high group. This is not in conformity with the literature, which states that social activities buffer/mitigate the negative effect of work-to-life negative spillover [11,16]. In a prospective cohort study conducted in Japan, level of social activities has been revealed as a protective factor for depressive symptoms, reporting people who engaged in physical exercises and local-community activities were less depressive than those who did not participate in social activities [16]. A longitudinal study showed that leisure time and physical exercises buffered the relationship between negative spillover and health outcomes [11]. 

This finding could be explained with reference to characteristics of people with high social activity satisfaction. People who are highly satisfied with social activities may have a low value for their work and are more likely to focus their life on activities other than work. They therefore may derive less meaning from their work, which results in increasing susceptibility of depressive symptoms when they have negative experiences in the workplace. Therefore, it should be noted that those who tend to be highly satisfied with social activities may become more vulnerable to depression if they have negative experiences at work. Based on these findings, organizations’ managers and policy makers may gain useful insights regarding employee mental health care planning, including with regard to depression screening and the development of interventions targeting employees vulnerable to depression. Providing them with interventions that can enhance the meaningfulness of work could also include alternative psychological management. For example, instead of supporting social activities outside work, organizations may support employees’ ‘job-crafting’, which is a self-initiated work design process that employees proactively make in the level of job demands and job resources in order to make their own work more satisfying and meaningful [33]. It may help employees to develop a sense of the meaningfulness of work in their workplace, rather than finding psychological well-being in places other than the workplace. This can be a new perspective on buffering negative work-to-life spillover and improving the mental health of working women.

Another possible explanation for this finding is that, for people with low social activity satisfaction, the only social activity may be economic activity, which may not greatly affect mental burdens even if there is a work-to-life negative spillover. Overall, we expanded existing literature which identifies moderating effects of social activities satisfaction on the relationship between work-to-life negative spillover and depressive symptoms, especially among working women in Korea.

The current study also has some limitations. First, this study used cross-sectional design, which limits causal inferences concerning work-to-life negative spillover and depressive symptoms. The possibility that depressive symptoms preceded work–life balance cannot be excluded. Further longitudinal study is needed to clarify whether work-to-life negative spillover precedes depressive symptoms. Second, this study assessed overall work and life experiences by using a self-report questionnaire. It is possible that there were recall biases (the unreliability of memory that makes interpretation of the results inconclusive) may have occurred. Third, although we used the CES-D scale to measure depressive symptoms, which is a widely used instrument for identification of depression with high reliability and validity, work-to-life negative spillover was measured with a non-validated assessing method, which calls for well-validated scales for statistical confirmation. Further, the lack of a theoretical or conceptual framework is one of the limitations of our study. Therefore, future studies may build more theories or conceptual frameworks in areas of work-to-life spillover, depressive symptoms, and social activities satisfaction in order to strengthen what is revealed through this study. 

Even so, the strengths of this study include its nationwide population-based data drawn from a representative sample of employed women in South Korea. Moreover, it is crucial to point out that, to our knowledge, this is the first study not only to find effects of work-to-life negative spillover on depression, but to examine the moderating effect of social activities satisfaction on the relationship between work-to-life spillover and depression. The unique finding that employees with low social activity satisfaction were revealed to be less depressive than those who with high social activity satisfaction, contrary to the expectations that social activities satisfaction buffers depression, is also a strength of this study. Based on the results of our study, effective strategies and policies for reducing work strain and the burden of domestic obligations is strongly recommended for a healthier work environment. Further, it is important to note that the effect of work-to-life negative spillover on depressive symptoms was different according to the degree of social activity satisfaction. It should therefore be considered when developing interventions for working women who experience work-to-life negative spillover, that a different approach could be implemented according to the degree or level of social activities satisfaction.

## 5. Conclusions

Overall, our findings indicate that working women who experience work-to-life negative spillover express high depressive symptoms. The results of our study also demonstrate that the impact of work-to-life negative spillover on working women’s depressive symptoms depends on the degree of satisfaction with social activities. It is our novel finding that women with low social activity satisfaction were revealed to be less depressive than those who with high social activity satisfaction. Based on the findings of our study, different approaches could be implemented according to the degree or level of social activity satisfaction when developing interventions for working women who experience work-to-life negative spillover. Effective strategies and policies for work-family compatibility and interventions aimed at reducing the work induced stress and depressive symptoms are also recommended.

## Figures and Tables

**Table 1:** Characteristics of socio-demographic data (N = 2869).

Variables		n	(%)
Age (Mean = 43.53, SD = 11.71)	<30	450	15.7%
	30–39	535	18.6%
	40–49	974	33.9%
	50–59	670	23.4%
	≥60	240	8.4%
Education	Elementary School	114	4.0%
	Middle School	155	5.4%
	High School	1049	36.6%
	Postgraduate	1551	54.1%
Marital status	Unmarried	726	25.3%
	Married	1845	64.3%
	Divorced or Separated	173	6.0%
	Bereaved	125	4.4%
Income satisfaction	Satisfied	796	27.7
	Moderate	1669	58.2
	Dissatisfied	404	14.1
Self-rated health	Good	1974	68.8
	Bad	895	31.2
Work-to-life negative spillover	Good	2679	93.4
Rewards and vitality in life	Bad	190	6.6
Recognition from family members	Good	2478	86.4
	Bad	391	13.6
Satisfaction with family life	Good	2465	85.9
	Bad	404	14.1
Influence of work on children	Good	1682	70.3
	Bad	709	29.7
Influence of work hours on fulfilling domestic obligations	Good	2021	70.4
	Bad	848	29.6
Influence of work schedule on family life	Good	2215	77.2
	Bad	654	22.8

**Table 2:** Association between work-to-life spillover and depressive symptoms.

		Non-Depressed Group		Depressed Group		
		n	(%)	n	(%)	*p*-Value
Total						
Age (y)	<30	362	12.6	88	3.1	0.04
	30–39	448	15.6	87	3.0	
	40–49	831	29	143	5.0	
	50–59	578	20.1	92	3.2	
	≥60	210	7.3	30	1.0	
Education	Elementary school	100	3.5	14	0.5	0.19
	Middle school	127	4.4	28	1.0	
	High school	904	31.5	145	5.1	
	Postgraduate	1298	45.2	253	8.8	
Income satisfaction	Satisfied	684	23.8	112	3.9	0.24
	Moderate	1407	49.0	262	9.1	
	Dissatisfied	338	11.8	66	2.3	
Marital status	Single	586	20.4	140	4.9	
	Married	1573	54.8	272	9.5	<0.001
	Divorced or Separated	155	5.4	18	0.6	
	Bereaved	115	4.0	10	0.3	
Self-rated health	Good	1674	58.3	300	10.5	0.75
	Bad	755	26.3	140	4.9	
Types of work-to-life negative spillover	Good	2286	79.7	393	13.7	<0.001
Rewards and vitality in life	Bad	143	5.0	47	1.6	
Recognition from family members	Good	2105	73.4	373	13.0	0.162
	Bad	324	11.3	67	2.3	
Satisfaction with family life	Good	2093	73.0	372	13.0	0.203
	Bad	336	11.7	68	2.4	
Influence of work on children	Good	1390	58.1	292	12.2	<0.001
	Bad	615	25.7	94	3.9	
Influence of work hours on fulfilling domestic obligations	Good	1783	62.1	238	8.3	<0.001
	Bad	646	22.5	202	7.0	
Influence of work schedule on family life	Good	1968	68.6	247	8.6	<0.001
	Bad	461	16.1	193	6.7	

**Table 3:** Association between work-to-life negative spillover and depressive symptoms.

	Depressive Symptoms			
	Crude Odds Ratio	(95% CI)	Adjusted Odds Ratio	(95% CI)
Types of work-to-life negative spillover				
Rewards and vitality in life	1.441 *	(0.83–2.51)	1.576 *	(0.88–2.82)
Recognition from family members	1.580	(0.94–2.66)	1.496	(0.94–2.38)
Satisfaction with family life	1.114	(0.71–1.93)	1.168	(0.71–1.94)
Influence of work on children	1.374 *	(1.07–1.77)	1.539 *	(1.19–2.00)
Influence of work hours on fulfilling domestic obligations	1.590 *	(1.12–2.27)	1.806 *	(1.28–2.54)
Influence of work schedule on family life	1.822 *	(1.26–2.63)	1.969 *	(1.37–2.83)

Note. Multiple logistic regression analysis was performed to examine the associations between work-to-life spillover and the depressive symptoms. * *p* < 0.05.

**Table 4:** Moderating effects of social activities satisfaction between work-to-life negative spillover and depression.

Variables	Model1	Model2	Model3	Model4
	β	β	β	β
Age	0.024	0.042	0.045	0.045
Education	−0.022	0.084	0.084	0.003
Marital status	0.140 ***	0.158 ***	0.153 ***	0.152 ***
Income	0.068 ***	0.079 **	0.053 **	0.052 **
Work-to-life negative spillover		0.079 ***	0.080 ***	0.064 **
Social activities satisfaction			0.055 **	−0.116
Work-to-life negative spillover * Social activities satisfaction				0.176 *
R^2^	0.031 ***	0.036 ***	0.039 ***	0.041 *
Adjusted R^2^		0.035	0.037	0.038
ΔR^2^		0.000	0.003	0.043

Note. * *p* < 0.05, ** *p* < 0.01, *** *p* < 0.001.
